# Identification of volatile biomarkers of *Trichomonas vaginalis* infection in vaginal discharge and urine

**DOI:** 10.1007/s00253-023-12484-6

**Published:** 2023-03-31

**Authors:** Ricardo Rubio-Sánchez, Rocío Ríos-Reina, Cristina Ubeda

**Affiliations:** 1 Servicio de Análisis Clínicos, Hospital Universitario Virgen de Valme, 41014 Seville, Spain; 2 Área de Nutrición y Bromatología, Departamento de Nutrición y Bromatología, Toxicología y Medicina Legal, Facultad de Farmacia, Universidad de Sevilla, 41012 Seville, Spain

**Keywords:** *Trichomonas vaginalis*, Trichomoniasis, Volatile organic compounds, Vaginal discharge, Urine, Diagnostic methods

## Abstract

**Abstract:**

Trichomoniasis, a disease caused by *Trichomonas vaginalis,* is the most common non-viral sexually transmitted infection worldwide. The importance of its diagnosis lies in its ease of transmission and the absence of symptoms in most cases, as occurs in men, which have a significant role as asymptomatic carriers. The most widely used diagnostic methods are the fresh examination of vaginal or urethral secretions and molecular techniques. However, as they have some disadvantages and, sometimes, low sensitivity, new trichomoniasis diagnostic methods are necessary. Volatile organic compounds in clinical samples are effective in the diagnosis of different diseases. This work aimed to study, for the first time, those present in vaginal discharge and urine of patients with *Trichomonas vaginalis* infection to look for volatile biomarkers. The results showed that volatile compounds such as 2-methyl-1-propanol and cyclohexanone could serve as biomarkers in vaginal discharge samples, as well as 2-octen-1-ol and 3-nonanone in urine. Moreover, 3-hydroxy-2,4,4-trimethylpentyl 2-methylpropanoate found in vaginal discharge, highly correlated to positive patients, is also highly related to urines of patients with trichomoniasis. The biomarkers described in this study might be a promising diagnostic tool.

**Key Points:**

• *The incidence of Trichomonas vaginalis infection is increasing*

• *Trichomonas vaginalis VOC study in vaginal discharge and urine was performed*

• *The identification of volatile biomarkers could allow a new diagnostic method*

## Introduction

*Trichomonas vaginalis*, a protozoan parasite of the human urogenital tract, is the causative agent of trichomoniasis, which is the most common non-viral sexually transmitted infection (STI) worldwide (Dittmer et al. 2021). In 2016, the World Health Organization (WHO) estimated the incidence of trichomoniasis to be 156 million new cases each year (Rowley et al. 2019).

On the one hand, the infection is usually asymptomatic in 10–50% of women, although the most frequent symptoms are pruritus, dysuria, vaginitis, vulvar erythema, and purulent vaginal discharge. Although rare, serious adverse outcomes of this STI include pelvic inflammatory disease, endometritis, infertility, cervical neoplasia, and an increased risk of infection with the human immunodeficiency virus (HIV) (Dittmer et al. 2021; Herath et al. 2021; Leitsch 2021; Mercer and Johnson 2018). A specific clinical sign of trichomoniasis is the strawberry cervix, characterized by pinpoint hemorrhagic lesions on the exocervix (Schwebke and Burgess 2004). During pregnancy, this STI can lead to premature destruction of membranes, preterm birth, low birth weight in infants, and neonatal death (Herath et al. 2021; Urbański et al. 2022). On the other hand, most men are asymptomatic carriers of the infection, although, in some cases, it can cause pruritus, dysuria, and prostatitis. Moreover, trichomoniasis accounts for 10–12% of non-gonococcal urethritis and increases the risk of developing prostate cancer (Dittmer et al. 2021; Herath et al. 2021; Leitsch 2021; Urbański et al. 2022; Galán et al. 2018). The high incidence of asymptomatic parasitosis in men makes them an endless vector, as they go undiagnosed and, thus, will continue to spread the infection (Soper 2004).

The most widely used diagnostic method in laboratories is the fresh examination of vaginal or urethral secretions, in which the visualization of motile trophozoites by microscopy has a specificity close to 100%, although with low sensitivity (45–60%). Microscopy has the advantage that it is a fast, easy to perform, and low-cost technique, but a temperature below 22 °C or a delay of 10–20 min in observation drastically decreases sensitivity, since the parasite rapidly loses its characteristic mobility. Otherwise, the culture of the sample in a liquid or semi-solid medium, mainly in Diamond’s broth, has been considered for many years the reference technique for *Trichomonas vaginalis* detection (Lawing et al. 2000). However, its sensitivity is also low (60–80%), and an incubation time of 2–7 days is required for parasite identification (Sherrard et al. 2014; Hirt and Sherrard 2015).

In recent years, molecular diagnostic methods based on the amplification of nucleic acids using polymerase chain reaction (PCR) have been described, which increases sensitivity concerning culture. Therefore, PCR is currently the reference technique. Another approved molecular technique detects 18S ribosomal RNA from *Trichomonas vaginalis*. Current techniques in the diagnosis of trichomoniasis through urine have low sensitivity, so a vaginal secretion sample is preferred (Lawing et al. 2000; Sherrard et al. 2014). Among the most current diagnostic methods are those based on nucleic acid hybridization and rapid tests, which can be used as point-of-care testing (POCT) (Herath et al. 2021; Schwebke and Burgess 2004; Galán et al. 2018; Hirt and Sherrard 2015), although they are not authorized on male samples. Furthermore, the only current diagnostic methods in men are culture and nucleic acid amplification tests (Sood et al. 2007; Nye et al. 2009; Hardick et al. 2003).

Volatile organic compounds (VOCs) are low molecular weight chemicals found in nature, also in multiple human matrices, such as breath, urine, feces, or sweat. The VOCs emitted by the human body vary according to sex, age, diet, and physiological state, among others, so each individual has a characteristic odor signature due to the combination of hundreds of odorous compounds that make up the volatilome. Different detectors can determine the presence of these VOCs in clinical samples, including gas chromatography-mass spectrometry (GC–MS), chemiluminescence, optical absorption spectroscopy, gas sensors, and electronic noses (e-noses) which are gas-based chemical detection devices. These last devices allow the detection of a specific set of VOCs present in a gas mixture, are much cheaper than GC–MS, and can be used as POCT because they are easy to use. E-nose allows the detection of disease-related volatiles and can be used to distinguish VOCs emitted in urine by patients with bacterial vaginosis (Sethi et al. 2013). Despite this, GC–MS is still considered the “golden standard” for VOC analysis although there are other alternatives such as selected ion flow tube-mass spectrometry (SIFT-MS), proton transfer reaction mass spectrometry (PTR-MS), or secondary electrospray ionization mass spectrometry (SESI-MS). In addition, other portable alternatives for VOC analysis are ion mobility spectrometers (IMS), among which time-of-flight IMS (ToF-IMS) stands out (Ratiu et al. 2020; Kunze-Szikszay et al. 2021).

Some metabolic disorders (phenylketonuria and maple syrup urine disease), various diseases (cancer, diabetes, Alzheimer's disease, and Crohn’s disease, among others), and other multiple infections alter the volatilome by producing new VOCs or modifying the proportion of those produced under normal conditions. Due to this, possible volatile biomarkers in clinical samples have been studied in recent years to diagnose different diseases quickly, in a noninvasive way, and in the early stages (Djago et al. 2021).

The importance of diagnosing *Trichomonas vaginalis* infection lies in its ease of transmission and the absence of symptoms in most cases. In addition, men’s role as asymptomatic carriers is increasing the incidence of this STI, making necessary new diagnostic methods for it. The use of urine as a biological sample would be a simple possibility considering that *Trichomonas vaginalis* affects the urogenital tract, and this parasite has been detected in the urine of 75% of women infected using standard methods. It is possible, therefore, that urethral colonization rates in women may be only 70 to 75% (Lawing et al. 2000; Kusdian and Gould 2014). Accordingly, the present study aimed to detect VOCs present in vaginal discharge and urine of patients with *Trichomonas vaginalis* infection to determine potential volatile biomarkers of trichomoniasis useful for early diagnosis of asymptomatic carriers, thus providing the possibility of developing a new advantageous methodology for the diagnosis of urogenital infection in patients and carriers mainly when using urine, as it is an easy biological sample to collect in both men and women.

## Materials and methods

### Samples

Two different types of samples, vaginal fluid and urine, were used in this study, which were collected between May 2020 and September 2021. Thirty vaginal discharge samples (15 positives for *Trichomonas vaginalis* and 15 negatives for this parasite) and 32 urine samples (17 from women with trichomoniasis and 15 from women without this parasite), from different women to avoid cross-contamination of VOCs, were collected. In addition, 3 vaginal discharge transport media without biological samples were also included in the study.

Vaginal discharge was collected and transported with ESwab (Copan Diagnostics, Italy), which combines a swab with Amies liquid. The sample was then transferred to the BD MAX UVE Sample Buffer Tube and tested on the BD MAX System using the BD MAX Vaginal Panel (Becton Dickinson, USA). The BD MAX Vaginal Panel uses real-time PCR for the amplification of specific DNA targets and fluorogenic target-specific hybridization probes to differentially detect bacterial vaginosis, *Candida* group, and *Trichomonas vaginalis* with a sensitivity of 93.2% and a specificity of 99.3% (BD MAX™ Vaginal Panel 2018). Instead, urine samples were collected in a BD Vacutainer tube without additives (Becton Dickinson, USA). One milliliter of each sample was subsequently analyzed by PCR as it is the reference technique, and the samples were classified according to their result. All collected samples were free of bacteria and *Candida* spp. After performing the PCR, all the samples were frozen at − 80 °C until analysis. For this, 2 mL of Amies liquid with vaginal discharge or 3 mL of urine from the selected patients was placed in a 20-mL headspace vial.

### Patients

The patients had a mean age of 36.8 years (range: 16–71). Table 1 shows the mean, 95% CI, and age range of each group, and it can be seen that there are no statistically significant differences between them.

**Table 1:** Age of the patients included in the study according to the sample used

	Negative vaginal discharge	Positive vaginal discharge	Negative urine	Positive urine
Arithmetic mean	34.7	34.7	36.3	40.9
95% CI	27.5–41.8	28.9–40.5	29.3–43.2	33.6–48.3
Range	16–64	19–51	21–62	21–71
*p*-value	0.450		0.636	

### Chemicals

Alkane standard mixture C10–C40, used for calculating Linear Retention Index (LRIs), was purchased from Fluka (Madrid, Spain). In addition, 4-methyl-2-pentanol (employed as internal standard), sodium chloride, and ethanol were of analytical quality and supplied by Merck (Darmstadt, Germany).

### Headspace-solid phase microextraction (HS-SPME)

HS-SPME extraction of the volatile compounds was performed for the analysis of the vaginal discharge and urine samples. Thus, once all the samples had been collected, the headspace vials were thawed at 4 °C for 12 h and mixed with 0.5 g of sodium chloride and 10 μL of 4-methyl-2-pentanol (0.75 mg/L). An MPS autosampler (Gerstel, Palo Alto, California) incubated the vial for 5 min at 45 °C with agitation at 300 rpm. Then, a 2 cm 50/30 μm Carboxen/DVB/PDMS SPME fiber (Supelco, Bellefonte, Pennsylvania) was exposed to the headspace of the vial for 30 min. After that, the fiber was desorbed in the injector in splitless mode for 180 s with the transfer line being at temperature 250 °C.

### Gas chromatography–mass spectrometry analysis

Analyses were performed in an Agilent 8890 gas chromatography system coupled to an Agilent 5977B Inert Plus quadrupole mass spectrometer with a Gerstel autosampler (Müllheim an der Ruhr, Germany). The capillary column employed was a J&W CPWax-57CB 50 m × 0.25 mm and 0.20 μm film thickness (Agilent, Santa Clara, CA, USA), flow rate of the helium was 1 mL/min, and the conditions were as follows: the oven temperature program started at 35 °C held for 4 min, followed by an increase to 220 °C at 2.5 °C/min held for 1 min. For both analyses, electron ionization mass spectra data were recorded from m/z 29–300 in scan mode with an ionization voltage of 70 eV.

All data from vaginal discharge and urine analyses were recorded using MSD ChemStation software (Agilent Technologies, Palo Alto, California), which was also used for peak area integration.

The NIST (National Institute of Standards and Technology) Mass Spectral Library was used for the identification of the volatile compounds and was carried out by comparing the mass spectra obtained from each molecule with the reference spectra of the NIST library. The identification was confirmed based on comparisons of the LRIs of standards with data from the literature. LRIs were calculated using the retention times of a series of n-alkanes analyzed under the same conditions as the samples. When only the software identification was possible, it was treated as tentatively identified. The data showed in this work were expressed as the relative area with respect to 4-methyl-2-pentanol (internal standard). The relative concentration was calculated by dividing the peak area of the target ion of each compound by the peak area of the target ion of the internal standard.

### Data treatment and chemometrics

The analysis of the volatile profiles for each dataset was carried out in several steps, starting with the integration of the peak areas of volatile compounds and the calculation of the relative concentrations according to the internal standard. A total of 134 and 125 volatile compounds were integrated and identified for urine and vaginal discharge datasets, respectively. Then, exploratory analyses using Principal Component Analysis (PCA) of each dataset, i.e., vaginal discharge and urine, were performed. Finally, a classification analysis was carried out for each dataset by Partial Least Squares Discriminant Analysis (PLS-DA), with the aim of feature selection by using the Variable Importance in the Projection (VIPs) obtained by each model. Data were autoscaled before modeling, and the total dataset was divided into a training and a test set for the external validation of the classification models: in the urine dataset, 24 samples were used as a training set (11 negatives and 13 positives) and 8 as a test set (4 negatives and 4 positives). In the case of vaginal discharge, 20 samples were used as a train set (10 negatives and 10 positives) and 10 in the test set (5 negatives and 5 positives).

All exploratory and classification analyses were performed using the PLS_Toolbox 7.9.5 (Eigenvector Research Inc., Wenatchee, WA, USA), working in a MATLAB environment (The Mathworks Inc., MA, USA, Version 2016b).

## Results

### Multivariate analysis

Due to the large number of volatile compounds determined in each sample type, 125 and 134 VOCs in vaginal discharge and urine, respectively, multivariate analysis was employed to define them with a higher responsibility in the differentiation among patients infected with *Trichomonas vaginalis* both in vaginal fluid and urine (positive samples) and non-infected patients (negative samples). Thus, each dataset was submitted to exploratory analyses using PCA.

**Fig. 1:**
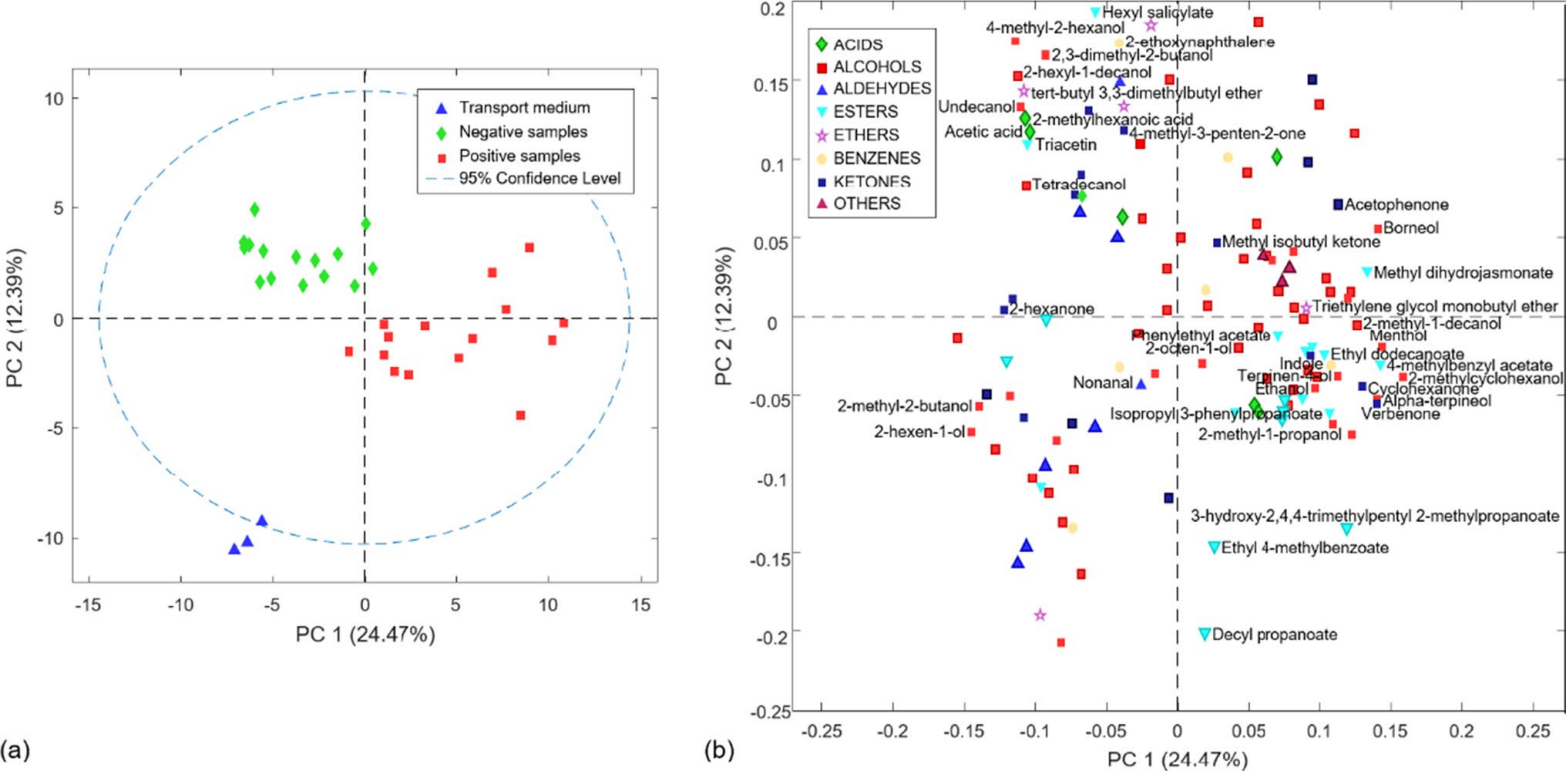
Score (**a**) and loading (**b**) plots of the first two PCs obtained by PCA based on the vaginal discharge dataset with the total number of volatile compounds detected

Regarding vaginal fluid samples, Fig. 1 shows the score (a) and loading (b) plots displaying the first two principal components (PCs) where the grouping of the samples according to the presence of *Trichomonas vaginalis* in the vaginal fluid can be observed. Thus, PC1, which explains 24.47% of the total variance, describes, on the negative side of PC1, the separation of negative samples and the transport medium from the positive samples on the positive side of PC1 (Fig. 1a). Looking at the loading plot (Fig. 1b), this separation was explained by a higher relation between almost all the identified aldehyde compounds and the negative samples, whereas most esters were more related to the positive ones. Furthermore, it can also be seen that PC2, which explains 12.39% of the total variance, describes the separation of all negative samples on the positive side from the transport medium on the negative side.

**Fig. 2:**
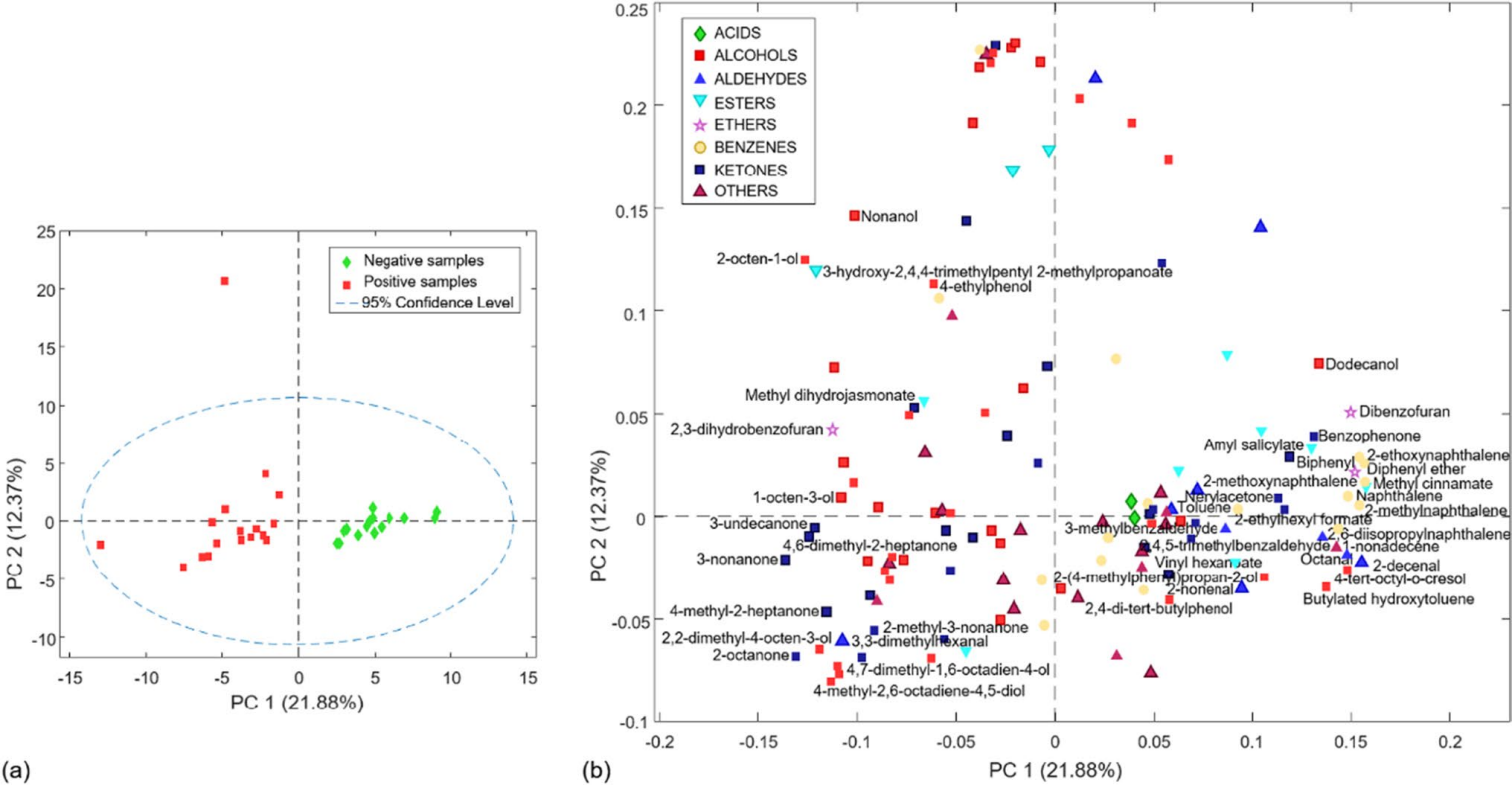
Score (**a**) and loading (**b**) plots of the first two PCs obtained by PCA on the urine dataset with the total number of volatile compounds detected

Concerning the urine dataset, Fig. 2 shows the score (a) and loading (b) plot displaying the first two PCs where the separation of the samples according to the presence of *Trichomonas vaginalis* in the urine can be clearly observed. Thus, PC1, which explains 21.88% of the total variance, describes the separation of all negative samples, on the positive side, related to the presence of acids and most aldehydes, from all positive samples on the negative side, which were more related to alcohols and esters according to the loadings (Fig. 2b).

After an exploratory analysis, classification analyses by PLS-DA were carried out, with the aim of selecting the main volatile compounds responsible for the differentiation of the negative samples from the positive ones that could be chosen as disease markers. Thus, as a supervised method, like PLS-DA, it can be performed for both classification and feature selection. Therefore, it could be considered that the analysis of the chromatographic profiles has been carried out in different steps, starting with the integration and identification of compounds, then the exploratory analyses using PCA previously discussed, and, finally, with the classification analyses for feature selection using PLS-DA models and the VIPs obtained through each model. A VIP score is a measure of the importance of a variable in the PLS-DA model that summarizes the contribution it makes to the model. VIP scores are scaled such that all predictors having a VIP greater than one are considered relevant. The higher the VIP, the higher the relevance is (Mehmood et al. 2012). For this reason, the selection of VIPs was applied to reduce the number of volatile compounds and look for markers.

PLS-DA models for vaginal fluid and urine datasets were built with three latent variables (accounting for 45.92% and 41.01% of explained variance, respectively) and satisfactory percentages of sensitivity, specificity, and error rate for calibration, cross-validation, and prediction (Table 2).

**Table 2:** Figures of merit obtained by the PLS-DA models for vaginal discharge and urine datasets built with three latent variables (accounting for 45.92% and 41.01% of explained variance, respectively)

Dataset	Model		Negative (%)	Positive (%)
PLS-DA vaginal exudate	Calibration	Sensitivity	100	100
		Specificity	100	100
		Error	0.00	0.00
	CV	Sensitivity	100	92.3
		Specificity	92.3	100
		Error	3.84	3.84
	Prediction	Sensitivity	100	100
		Specificity	100	100
		Error	0.00	0.00
PLS-DA urine	Calibration	Sensitivity	100	100
		Specificity	100	100
		Error	0.00	0.00
	CV	Sensitivity	100	100
		Specificity	100	100
		Error	0.00	0.00
	Prediction	Sensitivity	100	100
		Specificity	100	100
		Error	0.00	0.00

*Notes: CV*, cross-validation venetian blinds

A total of 41 and 45 volatile compounds showed VIPs > 1 in the PLS-DA models of vaginal discharge and urine, respectively; some of them were common between the two types of biological samples. Table 3 shows the relative area range of each volatile compound found in the vaginal discharge and urine samples of the patients considering the VIPs of both matrices, and Tables 4 and 5 show the specific VIPs for vaginal discharge and urine samples, respectively, and the VIP scores for each one.

**Table 3:** Relative area ranges and linear retention index (LRI) of the volatile organic compounds (VOCs) selected according to the VIPs scores (> 1) of the PLS-DA models carried out with each type of sample (vaginal discharge and urine)

			Vaginal discharge			Urine	
VOCs	LRI	Id	Negative	Positive	Transport medium	Negative	Positive
Alcohols							
ethanol	936	A	256–295	287–563	279	175–225	176–441
2-methyl-2-butanol	1017	A	6.75–53.9	2.61–51.3	54.9	ND	ND
2-methyl-1-propanol	1087	A	ND	0.34–14.8	ND	ND	ND
2,3-dimethyl-2-butanol	1102	A	12.2–30.2	1.78–25.2	ND	ND	ND
2,3-dimethyl-3-hexanol	1281	B	1.93–3.81	0.00–1.48	4.79	ND	ND
4-methyl-2-hexanol	1284	B	2.32–3.63	ND	ND	ND	ND
2-hexen-1-ol	1391	A	0.24–0.81	ND	1.27	ND	ND
2-methylcyclohexanol	1424	A	0.00–0.45	0.98–3.57	ND	ND	ND
1-octen-3-ol	1450	A	ND	ND	ND	0.28–1.98	0.26–3.67
octanol	1563	A	0.97–10.7	1.20–22.0	1.65	0.00–0.85	0.20–2.13
terpinen-4-ol	1603	A	ND	0.10–5.19	ND	0.00–4.09	0.00–6.33
2-octen-1-ol	1618	A	0.00–1.82	0.09–8.38	ND	ND	0.04–3.06
menthol	1647	A	0.00–0.79	0.09–2.38	ND	0.29–9.04	0.26–130
4-methyl-2,6-octadiene-4,5-diol	1667	B	ND	ND	ND	ND	0.00–5.32
nonanol	1668	A	0.14–2.46	0.37–2.08	0.24	0.17–0.65	0.16–2.17
2,2-dimethyl-4-octen-3-ol	1680	B	ND	ND	ND	0.38–0.87	0.10–55.1
4,7-dimethyl-1,6-octadien-4-ol	1684	B	ND	ND	ND	ND	0.00–4.97
alpha-terpineol	1705	A	ND	0.20–2.29	ND	0.00–0.24	0.00–1.78
borneol	1710	A	0.61–1.63	0.79–2.76	ND	0.21–0.48	0.00–7.23
2-methyl-1-decanol	1792	A	ND	0.00–0.75	ND	ND	ND
2-(4-methylphenyl)propan-2-ol	1855	A	ND	ND	ND	0.05–0.54	ND
undecanol	1874	A	0.00–1.00	ND	ND	ND	ND
butylated hydroxytoluene	1904	A	0.00–0.20	0.05–0.58	ND	5.55–22.3	1.27–7.80
dodecanol	1977	A	3.99–43.9	6.54–44.7	54.0	0.33–1.48	0.00–0.66
2-hexyl-1-decanol	2041	C	0.00–0.19	ND	ND	ND	ND
tetradecanol	2182	A	0.14–0.38	0.07–0.25	0.19	ND	ND
4-ethylphenol	2188	A	ND	ND	ND	0.00–0.34	0.00–2.43
2,4-di-tert-butylphenol	2269	A	21.2–73.8	18.5–101	163	7.54–93.1	11.1–48.7
4-tert-octyl-o-cresol	2384	B	ND	ND	ND	0.22–1.20	ND
Ketones							
methyl isobutyl ketone	996	A	21.6–74.8	18.7–90.1	25.3	32.2–73.9	21.0–65.6
2-hexanone	1062	A	4.43–20.2	1.01–17.4	16.8	ND	ND
4-methyl-3-penten-2-one	1114	A	0.00–18.4	0.00–1.89	ND	ND	ND
3-heptanone	1138	A	ND	ND	ND	1.86–5.41	0.00–4.03
4-methyl-2-heptanone	1198	A	6.83–17.5	4.68–26.4	20.3	0.00–0.78	0.00–9.15
4,6-dimethyl-2-heptanone	1234	A	0.41–1.02	0.26–0.93	0.97	0.00–0.20	0.00–0.68
2-octanone	1276	A	ND	ND	ND	0.00–1.15	0.00–7.37
cyclohexanone	1281	A	ND	0.49–7.37	ND	ND	ND
3-nonanone	1348	A	ND	ND	ND	ND	0.00–0.96
2-methyl-3-nonanone	1409	A	ND	ND	ND	0.00–0.12	0.00–0.88
3-undecanone	1559	A	ND	ND	ND	ND	0.00–0.75
acetophenone	1643	A	1.61–5.66	3.65–15.8	ND	0.84–2.05	0.34–58.8
verbenone	1718	A	ND	0.00–0.34	ND	ND	ND
nerylacetone	1852	A	0.22–0.82	0.16–1.04	ND	0.79–2.46	0.29–1.64
benzophenone	2366	C	0.45–1.13	0.43–0.64	0.81	1.93–4.55	1.04–4.00
Benzenes							
toluene	1017	A	ND	ND	ND	3.16–51.4	0.00–4.62
naphthalene	1729	A	ND	ND	ND	11.1–46.0	4.34–13.4
2-methylnaphthalene	1842	A	0.21–0.64	0.00–1.76	0.36	1.45–5.85	0.50–1.83
biphenyl	1983	A	ND	ND	ND	0.44–1.27	0.16–0.60
2-methoxynaphthalene	2186	B	ND	ND	ND	0.62–1.63	0.22–0.86
2-ethoxynaphthalene	2211	B	0.11–0.32	0.00–0.17	ND	0.65–1.62	0.26–0.96
2,6-diisopropylnaphthalene	2218	A	ND	ND	ND	0.04–0.45	0.00–0.05
indole	2345	C	0.00–57.1	0.75–96.9	11.6	ND	ND
Aldehydes							
octanal	1277	A	ND	ND	ND	0.38–0.68	ND
nonanal	1381	A	0.00–2.50	0.00–7.68	1.49	1.18–3.74	0.47–3.23
2-nonenal	1528	A	0.00–0.78	0.10–0.81	ND	0.08–0.21	0.00–0.24
2-decenal	1621	A	ND	ND	ND	0.21–0.42	ND
3-methylbenzaldehyde	1639	A	27.3–303	32.0–245	591	0.37–1.56	0.07–0.81
3,3-dimethylhexanal	1688	B	ND	ND	ND	ND	0.00–2.19
2,4,5-trimethylbenzaldehyde	1870	A	ND	ND	ND	2.99–8.66	0.00–8.21
Esters							
vinyl hexanoate	1311	B	ND	ND	ND	0.25–1.68	0.00–1.54
2-ethylhexyl formate	1440	B	ND	ND	ND	0.07–0.91	0.00–0.74
ethyl 4-methylbenzoate	1784	B	0.07–0.27	0.16–0.62	0.38	ND	ND
phenylethyl acetate	1810	A	ND	0.00–3.95	ND	ND	ND
4-methylbenzyl acetate	1826	B	0.00–0.28	0.00–3.41	ND	ND	ND
ethyl dodecanoate	1839	A	0.00–0.18	0.00–1.58	ND	ND	ND
isopropyl 3-phenylpropanoate	1866	B	ND	0.00–0.37	ND	ND	ND
3-hydroxy-2,4,4-trimethylpentyl 2- methylpropanoate	1873	A	0.36–4.94	2.93–15.4	9.86	0.18–0.68	0.40–1.21
decyl propanoate	1949	C	0.00–0.75	0.34–1.38	1.46	ND	ND
methyl cinnamate	2082	A	ND	ND	ND	0.28–0.74	0.11–0.37
triacetin	2084	A	0.00–3.18	0.00–1.32	ND	ND	ND
amyl salicylate	2106	A	ND	ND	ND	0.64–2.25	0.00–1.14
hexyl salicylate	2206	A	0.26–0.81	0.22–0.36	ND	1.07–3.11	0.47–4.79
methyl dihydrojasmonate	2258	A	0.00–0.20	0.03–1.16	ND	ND	0.00–0.60
Acids							
acetic acid	1456	A	0.00–118	0.00–26.4	ND	0.38–2.10	0.00–4.28
2-methylhexanoic acid	1846	C	0.00–1.96	ND	ND	ND	ND
Ethers							
tert-butyl 3,3-dimethylbutyl ether	1594	B	2.16–30.8	0.72–16.3	1.40	ND	ND
diphenyl ether	2004	A	0.00–0.28	0.10–0.15	ND	0.56–2.13	0.19–0.77
triethylene glycol monobutyl ether	2209	B	0.07–0.67	0.26–3.02	ND	ND	ND
dibenzofuran	2236	C	ND	ND	ND	0.33–0.66	0.15–0.49
2,3-dihydrobenzofuran	2321	C	ND	ND	ND	ND	0.00–1.30
Alkanes							
1-nonadecene	2044	C	ND	ND	ND	0.54–2.79	ND

Notes: The identification of VOCs was carried out by comparing each mass spectra obtained with the mass spectral database (NIST), and LRI agreed with data from the literature (Pherobase: www.pherobase.com, NIST Mass Spectrometry Data Center: https://webbook.nist.gov, LRI and Odour database: http://www.odour.org.uk/lriindex.html). *ND*, non-detected. Relative areas are multiplied by 100. A: mass spectrum agreed with the mass spectral database, and LRI agreed with the literature data. B: mass spectrum agreed with mass spectral database, but there is no LRI in a polar column reported in the literature. C: mass spectrum agreed with mass spectral database R.Match ˃ 800 but not with LRI in the literature

**Table 4:** Score values of the variable importance in projection (VIP) index obtained by the PLS-DA classification model made with vaginal discharge dataset

VOCs vaginal discharge	Chemical family	VIP scores	Related to
2-methylcyclohexanol	Alcohols	3.99	P
cyclohexanone	Ketones	3.65	P
alpha-terpineol	Alcohols	3.52	P
3-hydroxy-2,4,4-trimethylpentyl 2-methylpropanoate	Esters	3.51	P
menthol	Alcohols	2.97	P
borneol	Alcohols	2.80	P
decyl propanoate	Esters	2.74	P
terpinen-4-ol	Alcohols	2.56	P
verbenone	Ketones	2.23	P
2-methyl-1-decanol	Alcohols	2.07	P
triethylene glycol monobutyl ether	Ethers	1.90	P
2-methyl-1-propanol	Alcohols	1.87	P
methyl dihydrojasmonate	Esters	1.86	P
4-methylbenzyl acetate	Esters	1.81	P
octanol	Alcohols	1.77	P
ethanol	Alcohols	1.71	P
nonanal	Aldehydes	1.48	P
phenylethyl acetate	Esters	1.48	P
2-octen-1-ol	Alcohols	1.46	P
isopropyl 3-phenylpropanoate	Esters	1.38	P
ethyl dodecanoate	Esters	1.33	P
acetophenone	Ketones	1.23	P
ethyl 4-methylbenzoate	Esters	1.19	P
indole	Benzenes	1.17	P
4-methyl-2-hexanol	Alcohols	5.03	N
2,3-dimethyl-3-hexanol	Alcohols	4.45	N
2-hexen-1-ol	Alcohols	4.17	N
2-hexyl-1-decanol	Alcohols	3.97	N
undecanol	Alcohols	2.28	N
2,3-dimethyl-2-butanol	Alcohols	2.26	N
tetradecanol	Alcohols	2.01	N
tert-butyl 3,3-dimethylbutyl ether	Ethers	1.98	N
2-methylhexanoic acid	Acids	1.96	N
2-ethoxynaphthalene	Benzenes	1.74	N
hexyl salicylate	Esters	1.73	N
4-methyl-3-penten-2-one	Ketones	1.41	N
triacetin	Esters	1.34	N
acetic acid	Acids	1.19	N
2-hexanone	Ketones	1.09	N
methyl isobutyl ketone	Ketones	1.08	N
2-methyl-2-butanol	Alcohols	1.05	N

*Notes: P*, positive samples (infected people); *N*, negative or control samples (healthy people)

**Table 5:** Score values of the variable importance in projection (VIP) index obtained by the PLS-DA classification model made with urine dataset

VOCs urine	Chemical family	VIP scores	Related to
3-nonanone	Ketones	2.49	P
3-hydroxy-2,4,4-trimethylpentyl 2-methylpropanoate	Esters	2.28	P
2-octen-1-ol	Alcohols	1.85	P
3-undecanone	Ketones	1.85	P
4,6-dimethyl-2-heptanone	Ketones	1.72	P
octanol	Alcohols	1.58	P
methyl dihydrojasmonate	Esters	1.46	P
2-octanone	Ketones	1.39	P
nonanol	Alcohols	1.35	P
2-methyl-3-nonanone	Ketones	1.34	P
1-octen-3-ol	Alcohols	1.33	P
2,3-dihydrobenzofuran	Ethers	1.28	P
2,2-dimethyl-4-octen-3-ol	Alcohols	1.25	P
4-methyl-2-heptanone	Ketones	1.15	P
octanal	Aldehydes	4.71	N
2-decenal	Aldehydes	4.68	N
4-tert-octyl-o-cresol	Alcohols	4.40	N
1-nonadecene	Alkanes	4.10	N
toluene	Benzenes	3.65	N
butylated hydroxytoluene	Alcohols	3.48	N
2-nonenal	Aldehydes	3.14	N
methyl cinnamate	Esters	2.89	N
2-methoxynaphthalene	Benzenes	2.82	N
2-ethoxynaphthalene	Benzenes	2.78	N
biphenyl	Benzenes	2.76	N
2-methylnaphthalene	Benzenes	2.68	N
diphenyl ether	Ethers	2.50	N
naphthalene	Benzenes	2.49	N
2,6-diisopropylnaphthalene	Benzenes	2.42	N
dibenzofuran	Ethers	2.38	N
amyl salicylate	Esters	2.23	N
nerylacetone	Ketones	1.96	N
2-(4-methylphenyl)propan-2-ol	Alcohols	1.96	N
2,4,5-trimethylbenzaldehyde	Aldehydes	1.91	N
dodecanol	Alcohols	1.73	N
3-heptanone	Ketones	1.61	N
3-methylbenzaldehyde	Aldehydes	1.60	N
2-ethylhexyl formate	Esters	1.45	N
benzophenone	Ketones	1.41	N
2,4-di-tert-butylphenol	Alcohols	1.20	N
vinyl hexanoate	Esters	1.17	N
4-ethylphenol	Alcohols	1.03	N
4-methyl-2,6-octadiene-4,5-diol	Alcohols	1.03	N
4,7-dimethyl-1,6-octadien-4-ol	Alcohols	1.02	N
3,3-dimethylhexanal	Aldehydes	1.01	N

*Notes: P*, positive samples (infected people); *N*, negative or control samples (healthy people)

### Analysis of the VOCs from vaginal discharge

From the 41 VIPs obtained from the PLS-DA model made with the vaginal discharge dataset, it could be seen in Table 4 that the majority of groups of volatile compounds belonged to the families of alcohols (18), esters (10), and ketones (6). Moreover, 24 of them, out of 41 VIPs, were more related to positive samples. Eight volatile compounds were detected in the infected samples which were not found in the negative ones (Table 3). Among them, 2-methyl-1-propanol, terpinen-4-ol, alpha-terpineol, and cyclohexanone were identified in all patients infected with the parasite (Table 3). This agreed with the high VIP scores accounted for them (Table 4); the last three were among the 10 most related to the infected patients’ compounds.

It was also observed that positive samples presented higher amounts of primary alcohols (ethanol, 2-methyl-1-propanol, octanol, and 2-octen-1-ol) and secondary alcohols (2-methylcyclohexanol and menthol) than negative samples. The quantity of ethanol produced was the most remarkable (Table 3).

Furthermore, regarding the ketones, cyclohexanone and acetophenone were found in the vaginal discharge of patients with *Trichomonas vaginalis*. Cyclohexanone was the volatile compound with the second highest VIP score, only preceded by 2-methylcyclohexanol, and it was not detected in negative nor in urine samples. Acetophenone was detected in both types of samples, negative and positive, but it seemed to be in higher concentration in the latter one, something which also occurs in urine samples (Table 3).

As can be observed in Fig. 1, the positive vaginal discharge samples were also highly correlated to esters; thus, 8 esters presented VIP scores superior to 1 in these samples (Table 4). Among them, 3-hydroxy-2,4,4-trimethylpentyl 2-methyl propanoate and decyl propanoate ranked among the top 10 potential biomarkers of the infection. Apart from them, ethyl and acetate esters were detected within those with a VIP score above 1.

In addition to the mentioned compounds, some alcohols were found in the control patients (negative samples) but not in the patients with trichomoniasis (positive samples). Specifically, seven accounted for the higher VIP scores, more related to the samples without trichomoniasis (Table 4). These alcohols were sorted in order from higher to lower VIP score: 4-methyl-2-hexanol, 2,3-dimethyl-3-hexanol, 2-hexen-1-ol, 2-hexyl-1-decanol, undecanol, 2,3-dimethyl-2-butanol, and tetradecanol. Among them, 4-methyl-2-hexanol was present in all the negative samples, but not in the positive ones or the transport medium (Table 3).

### Analysis of the VOCs from urine samples

Although the detection of *Trichomonas vaginalis* was carried out in the vaginal discharge, the VOCs present in the urine samples of patients with trichomoniasis were also analyzed to search for biomarkers that characterize the presence of this parasite also in the urinal tract. Thus, among the 45 VIPs selected after the multivariate analysis (Table 5), the main chemical groups were alcohols (13), ketones (9), and benzenes (7) which were much more abundant in urine in contrast to vaginal discharge (Table 3).

Among the volatile compounds with VIP scores > 1 found in urine samples, 5 of them were also present in vaginal discharge: octanol, 2-octen-1-ol, 3-hydroxy-2,4,4-trimethylpentyl 2-methylpropanoate, methyl dihydrojasmonate, and 2-ethoxynaphthalene (Table 3). All of them, excluding 2-ethoxynaphtalene, were highly correlated with the positive patients, among them, the 7 with the highest VIP scores (Table 5). These compounds were also positively correlated with the patients with trichomoniasis in the vaginal discharge samples; however, only 3-hydroxy-2,4,4-trimethylpentyl 2-methylpropanoate got a remarkable VIP score (i.e., higher of 3), the fourth highest related to positive samples.

As mentioned above, a higher number of ketones were detected in urine with respect to vaginal discharge. Several of them were not found in the urine of healthy patients, or they were found in a very low quantity, among which 3-nonanone, 3-undecanone, and 4,6-dimethyl-2-heptanone stand out, being among the five highest VIP scores related to positive patients (Table 5).

## Discussion

### Analysis of the VOCs from vaginal discharge

The higher presence of primary and secondary alcohols in positive samples analyzed from vaginal discharge could be explained by the fact that *Trichomonas vaginalis* might produce them, according to the literature. Thus, their presence agrees with Sutak et al. (2012), who reported that *Trichomonas vaginalis* produces ethanol and other secondary alcohols when it grows in a medium with maltose, in a reaction catalyzed by secondary alcohol dehydrogenase (S-ADH). This enzyme catalyzes the reversible reduction of ketones into secondary alcohols, which would justify the higher production of 2-methylcyclohexanol and menthol in the positive samples observed in our study. Furthermore, just as this enzyme reduces acetaldehyde to ethanol, it can also reduce other aldehydes to primary alcohols, which would justify the production of 2-methyl-1-propanol, octanol, and 2-octen-1-ol by this parasite (Smutná et al. 2022; Kleiner and Johnston 1985).

The notable amount of esters found in the vaginal discharge of infected women can also be due to the parasite metabolism. Thus, the energy metabolism of *Trichomonas vaginalis* is based on the anaerobic fermentation of glucose, since hydrogenosomes lack an electron transport chain and, therefore, this parasite cannot obtain adenosine triphosphate (ATP) through oxidative phosphorylation (Dittmer et al. 2021; Schneider et al. 2011). Glucose is obtained mainly from the breakdown of glycogen present in vaginal epithelial cells, the distal urethra, or polysaccharides accumulated intracellularly in the parasite (Dittmer et al. 2021). Glucose is metabolized by glycolysis in the cytosol, giving rise to pyruvate and malate (Leitsch 2021). Pyruvate is oxidatively decarboxylated by pyruvate:ferredoxin oxidoreductase (pyruvate synthase) to acetyl-CoA and CO_2_, also producing, indirectly, molecular hydrogen. The acetyl-CoA obtained is converted to acetate by acetate:succinate-CoA transferase, in a succinate-dependent reaction, and indirectly produces ATP (Tachezy et al. 2022; Beltrán et al. 2013). The production of acetyl-CoA could justify the presence of acetate esters formed by enzymatic condensation of acetyl-CoA and the corresponding alcohols. Likewise, ethyl esters such ethyl 4-methylbenzoate could be formed from the esterification of ethanol and the corresponding acid. This is not surprising since, as mentioned above, this alcohol seemed to be the most significantly produced compound when *Trichomonas vaginalis* was present (Tables 3 and 4).

As previously reported, 4-methyl-2-hexanol was present in all the negative samples, but not in the positive ones or the transport medium. Maybe this compound is produced by the microbiota present in the vaginal tract, due to its absence in the transport medium, and metabolized/degraded by *Trichomonas vaginalis*. Other alcohols presented in the transport medium, such as 2,3-dimethyl-3-hexanol and 2-hexen-1-ol, seemed to be degraded in all vaginal fluids; however, when *Trichomonas vaginalis* was present, they showed a consumption/degradation to a greater extent due to the lower values observed (Table 3).

### Analysis of the VOCs from urine samples

As previously reported, among the volatile compounds that appear in both vaginal discharge and urine and were related to positive samples, i.e., 3-hydroxy-2,4,4-trimethylpentyl 2-methylpropanoate, octanol, 2-octen-1-ol, and methyl dihydrojasmonate, only the first one got a remarkable VIP score (i.e., higher than 3). However, it is important to consider that the biological samples are very different, and the parasite may degrade/metabolize the substrates available in each medium, urine or vaginal discharge, in a different way or show a different performance, taking into account their abundance in the medium. Therefore, this result could point to these compounds as potential biomarkers present in vaginal discharge and urine.

In addition, in both positive vaginal fluid and positive urine, higher amounts of octanol and 2-octen-1-ol, primary alcohols, were observed. Although octanol was present in the vaginal discharge transport medium and urine of healthy patients (Drabińska et al. 2021), its amount was higher in both positive samples. In contrast, 2-octen-1-ol was not detected in the transport medium, and its presence naturally in urine has not been documented, but it was also higher in samples from patients diagnosed with trichomoniasis. In the case of 2-ethoxynaphtalene, it was found both in urine and vaginal discharge more related to negative patients, which could indicate that this compound is consumed or degraded by *Trichomonas vaginalis*.

As previously mentioned, a large number of ketones were detected in the positive samples whose production, according to other authors, may be due to a reversible oxidation of secondary alcohols by S-ADH activity in *Trichomonas vaginalis* (Kleiner and Johnston 1985).

Regarding the aldehydes, *Trichomonas vaginalis* might be able to consume octanal in urine, as it is an aldehyde found in healthy patients (Drabińska et al. 2021), but it is not detected in positive samples, ranking first in the VIPs scores related to healthy or negative patients. This decrease is consistent with the octanol production discussed above, as the S-ADH enzyme can reduce octanal (Sutak et al. 2012). In addition, 2-decenal was not detected in positive urine, unlike in negative urine; therefore, it may be that *Trichomonas vaginalis* consumes this aldehyde or that the presence of the parasite prevents the growth of non-pathogenic microbiota that in healthy patients produces 2-decenal.

The presence of toluene has been reported naturally in urine (Drabińska et al. 2021), so its lower concentration in the presence of *Trichomonas vaginalis*, even undetectable in most samples, could be due to the higher consumption/degradation caused by the parasite. Although the degradation of benzenic compounds has not been documented in parasites, some anaerobic bacteria degrade toluene by binding to fumarate and forming (R)-benzylsuccinate in a reaction catalyzed by the enzyme glycyl radical benzylsuccinate synthase (Rajamanickam and Baskaran 2017; Vogt et al. 2019). In this context, regarding the results shown in Table 3, it was observed that toluene degradation occurred in urine samples, and since fumarate is a secondary product of the urea cycle, we hypothesize that *Trichomonas vaginalis* might degrade toluene by this mechanism.

Diagnostic tests are only performed on women in most laboratories because most men are asymptomatic carriers of the infection. For this reason, all the patients included in this study were women. Potential volatile biomarkers of *Trichomonas vaginalis* infection identified in urine could be used as POCT, facilitating rapid diagnosis, especially useful in asymptomatic carriers such as men.

In conclusion, the volatile profile of the samples used in the diagnosis of trichomoniasis had not been analyzed until now, so the analysis of VOCs in vaginal discharge and urine represents an important step in understanding the metabolism of *Trichomonas vaginalis*, and in the search of new diagnostic methods. Based on our results, 2-methyl-1-propanol and cyclohexanone are compounds that were only detected in parasitized vaginal discharge and, as well as 2-methylcyclohexanol and 3-hydroxy-2,4,4-trimethylpentyl 2-methylpropanoate, could serve as volatile biomarkers for *Trichomonas vaginalis* in these samples. Moreover, 2-octen-1-ol and 3-nonanone could be considered volatile biomarkers of this parasite in urine since they were only detected in the parasitized samples. Furthermore, 3-hydroxy-2,4,4-trimethylpentyl 2-methylpropanoate found in vaginal discharge and highly correlated to positive patients was also highly related to their urine samples. Considering that the high prevalence of asymptomatic *Trichomonas vaginalis* infection in men makes them the predominant carriers of the disease, easily transmitting this STI, revealing potential volatile biomarkers of trichomoniasis in urine could be a promising diagnostic technique. These volatile compounds could be detected by electronic noses, thus being a valuable tool in daily clinical practice as they are cheap and can be used as POCT. However, future research analyzing the urine of males must be done to find a new method to rapidly diagnose asymptomatic carriers to stop the transmission of this STI.

## Data Availability

All data can be provided by the corresponding author upon request.
